# [Retracted] Renal denervation attenuates cardiac hypertrophy in spontaneously hypertensive rats via regulation of autophagy

**DOI:** 10.3892/mmr.2025.13663

**Published:** 2025-08-26

**Authors:** Jionghua Huang, He Huang, Wei Pan, Dejin Ou, Wenjun Dai, Yuhui Lin, Jinlei Wu, Wenjie Xie, Ximing Chen

Mol Med Rep 16: 2023–2029, 2017; DOI: 10.3892/mmr.2017.6790

Following the publication of the above paper, a concerned reader drew to the Editor's attention that data featured in Figs. 1B, 3 and 4 had previously appeared in different contexts in three papers published in other journals (two papers in *PLoS One*, and one article in *Cardiovascular Journal of Africa*) that had been written by the same research group, or which contained members thereof. Moreover, the two articles featured in *PLoS One* have subsequently been retracted on account of the misassembly of various of the figures.

An independent investigation of the data in this paper performed by the Editorial Office confirmed these concerns; therefore, the Editor of *Molcular Medicine Reports* has determined that this paper should be retracted from the Journal on account of a lack of confidence in the presented data. The Editor regrets any inconvenience that has been caused to the readership of the Journal.

